# DTDNet: Dynamic Target Driven Network for pedestrian trajectory prediction

**DOI:** 10.3389/fnins.2024.1346374

**Published:** 2024-04-30

**Authors:** Shaohua Liu, Jingkai Sun, Pengfei Yao, Yinglong Zhu, Tianlu Mao, Zhaoqi Wang

**Affiliations:** ^1^School of Electronic Engineering, Beijing University of Posts and Telecommunications, Beijing, China; ^2^Beijing Key Laboratory of Mobile Computing and Pervasive Device, Institute of Computing Technology, Chinese Academy of Sciences, Beijing, China; ^3^School of Computer Science and Technology, University of Chinese Academy of Science, Beijing, China

**Keywords:** multimodal trajectory prediction, pedestrian intention prediction, multi-precision motion prediction, multi-task neural network, trajectory endpoint prediction

## Abstract

Predicting the trajectories of pedestrians is an important and difficult task for many applications, such as robot navigation and autonomous driving. Most of the existing methods believe that an accurate prediction of the pedestrian intention can improve the prediction quality. These works tend to predict a fixed destination coordinate as the agent intention and predict the future trajectory accordingly. However, in the process of moving, the intention of a pedestrian could be a definite location or a general direction and area, and may change dynamically with the changes of surrounding. Thus, regarding the agent intention as a fixed 2-d coordinate is insufficient to improve the future trajectory prediction. To address this problem, we propose Dynamic Target Driven Network for pedestrian trajectory prediction (DTDNet), which employs a multi-precision pedestrian intention analysis module to capture this dynamic. To ensure that this extracted feature contains comprehensive intention information, we design three sub-tasks: predicting coarse-precision endpoint coordinate, predicting fine-precision endpoint coordinate and scoring scene sub-regions. In addition, we propose a original multi-precision trajectory data extraction method to achieve multi-resolution representation of future intention and make it easier to extract local scene information. We compare our model with previous methods on two publicly available datasets (ETH-UCY and Stanford Drone Dataset). The experimental results show that our DTDNet achieves better trajectory prediction performance, and conducts better pedestrian intention feature representation.

## 1 Introduction

Trajectory prediction is an essential research area that has various applications in autonomous driving (Bennewitz et al., 2005; Ma et al., 2019; Chandra et al., 2020), robot navigation (Rasouli et al., 2019), and surveillance systems (Oh et al., 2011; Sultani et al., 2018). For instance, in autonomous driving, vehicles need to estimate the future movements of pedestrians to avoid collisions and plan a safe driving path.

One of the basic challenges for trajectory prediction is to analyze the pedestrian future intention in the changing context, such as whether the pedestrian intends to cross the road before or after a car passes. This analysis can provide a useful information for trajectory prediction. Recently, some works have considered the agent intention prediction in the trajectory prediction task, such as PECNet (Mangalam et al., 2020), TNT (Zhao et al., 2021), DenseTNT (Gu et al., 2021), and so on. However, these methods simplify the problem by assuming that the agent intention endpoint, which reveals the agent movement intention, remains constant during the prediction range.

**Figure 1:**
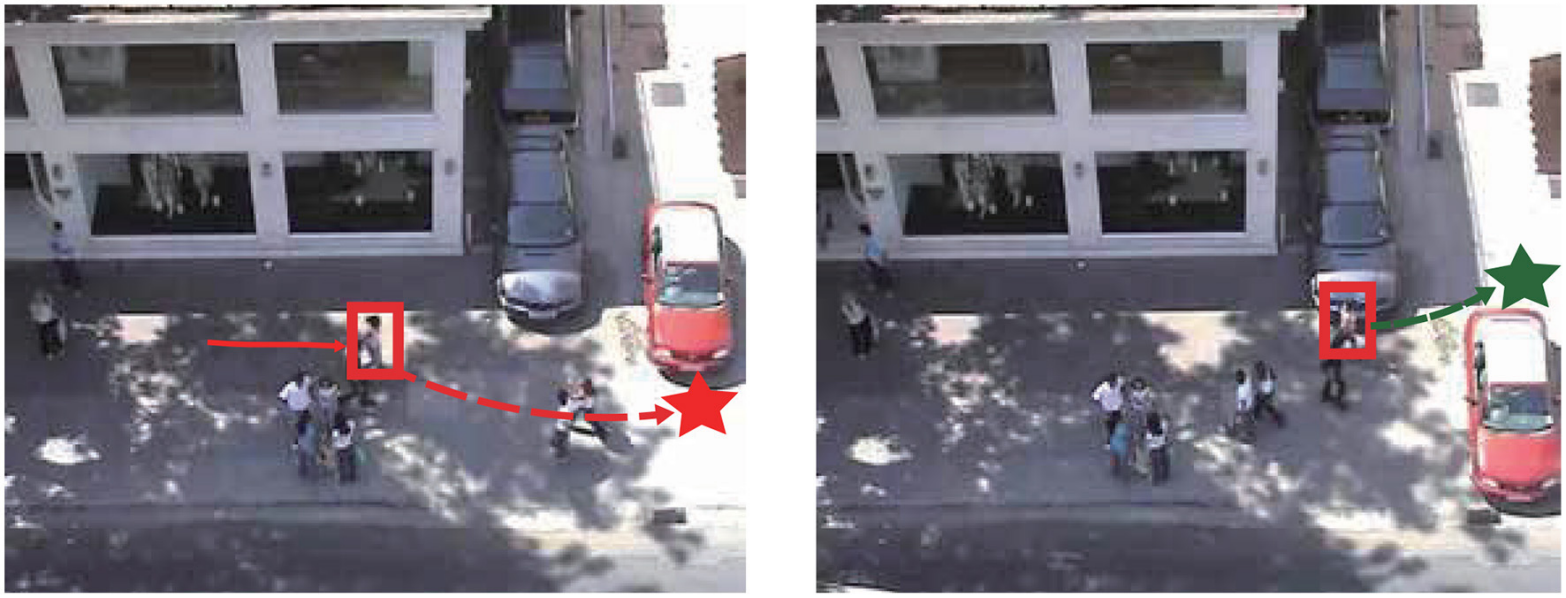
Dynamic change of the pedestrian intention.

In fact, predicting the endpoint coordinates of pedestrians is a very challenging task. Pedestrians will dynamically adjust their intent endpoint coordinates in respond to the change of scene information in different regions. As shown in Figure 1, the pedestrian in the red frame is the target pedestrian. In the left image, the vehicle on the right is parked at the upper right of the image and has no tendency to move forward. At this time, the short-term movement target of the pedestrian is the red star below the vehicle. However, during the movement of the pedestrian, the vehicle starts to move forward, blocking the original movement target of the pedestrian. Due to environmental changes, pedestrians must change their original intention and move toward the green star at the upper right. It is important to dynamically analyze the pedestrian's intent coordinate by combining the pedestrian's motion state and scene characteristics.

In addition, when modeling the future intention of the pedestrian, existing methods generally use the multi-layer perceptrons (MLPs) to predict a 2-d coordinate as the intention feature. Huang et al. (2021) models the intention with a Mutable Intention Filter to address the drift in long-term pedestrian trajectory prediction, and its experiment demonstrates the goal prediction is changing during the prediction process. But there are limitations in the work. Firstly, this work assumes that all targets are located at the scene edges, which is unrealistic. And it models the intention with specific 2-D locations. The pedestrian's movement intention information should not be modeled as a specific physical coordinate, and the observable coordinate cannot fully represent the pedestrian's intention to help predict the future trajectory as in Figure 1.

In this paper, we model the intention as features that combine both fine-precision destination and coarse-precision region representation, and could be dynamically changed in the prediction process, consider the dynamic changing caused by environment and pedestrian. To extract a feasible dynamic intention feature, we propose a multi-precision pedestrian intention analysis module, which dynamically predicts intent from the scene information and history trajectory. We generate the coarse-precision coordinate from the history trajectory, then we use the scene heatmap and the coarse-precision coordinate to calculate the local dynamic feature. By combining the local dynamic feature and the coarse-precision coordinate, we predict agent intention feature as an assistance to predicting the future trajectory. In addition, three sub-tasks including prediction of coarse-precision endpoint coordinate, fine-precision endpoint coordinate and scene sub-regions scoring are proposed to help training the feasible dynamic agent intent extraction module.

We propose Dynamic Target Driven Network for pedestrian trajectory prediction (DTDNet). First, we use a motion pattern encoding module to extract movement patterns from pedestrian historical trajectories. After that, we use multi-precision pedestrian intention analysis module to extract the feasible intention based on multi-precision feature input. At the same time, multi-precision intention analysis sub-tasks are introduced to aid pedestrian intent information extraction. Finally, a pedestrian trajectory decoding module based on the CVAE generation framework combines pedestrian movement patterns and scene information to predict pedestrian intent coordinates dynamically. The contributions of this paper are as follows:

1. We discuss the dynamic changing attribute of pedestrian intention prediction process, and propose a novel module to extract the dynamic intention feature accordingly. This module encodes the pedestrian future intention at each time steps iteratively with scene information, and we propose a multi-task structure to aid the feature learning process with three related subtasks.
2. We propose a novel multi-precision pedestrian trajectory data representation method to estimate the multi-precision intention, including three aspects: coarse-precision coordinates, fine-precision coordinates, and local scene information.
3. We design a new trajectory prediction model DTDNet, which conducts the prediction with dynamic intention modeling and multi-precision history data. Qualitative and quantitative experiments show that this model outperforms current methods and predicts endpoint coordinates closer to the future endpoint.

## 2 Related work

### 2.1 Trajectory prediction

Early researches on trajectory prediction are based on hand-craft rules and energy potentials. Helbing and Molnar (1995) model the force between pedestrians by attractive force and repulsive force. However, with the limitation of the hand-craft functions, the previous approaches cannot model the complicated interactions in crowded scenarios. Trajectory prediction is a time series prediction task, many data-driven methods (Oliveira et al., 2021; Zhang et al., 2022) have been proposed to solve this problem in recent years. Alahi et al. (2016) propose one of the earliest deep learning models for trajectory prediction, which uses a grid-based “social pooling” layer to aggregate the hidden state of the pedestrians in the neighborhood. Gupta et al. (2018) also use the pooling-based method and propose a “pooling module” to share information of all the pedestrians in the whole scene. Vemula et al. (2018) and Kosaraju et al. (2019) introduce the attention mechanism to assign different importance to different agents. Recent works (Huang et al., 2019; Hu et al., 2020; Mohamed et al., 2020; Tao et al., 2020) are all graph-based methods that use graph neural networks to model the interactions among the pedestrians.

### 2.2 Human-scene interaction

Pedestrian motion is not only affected by surrounding pedestrians, but the layout features of the scene also limit the movement space of pedestrians. Therefore, effectively extracting scene information plays a crucial role in trajectory prediction. Some works (Vemula et al., 2018; Huang et al., 2019) use VGGNet to encode a large scene's complete overhead image information. The model can learn any scene information and use the visual attention mechanism to assign important spatial regions to pedestrians. To incorporate scene category information, Yao et al. (2021) use a semantic segmentation model to process scene pictures. Pixel-level scene category information can be obtained by using semantic segmentation information. However, this method still has ambiguous information and does not know whether pedestrians in this category could move forward. Wang et al. (2022) proposed a heat map construction method based on historical trajectory statistics and used the GLU module to model scene information continuity.

### 2.3 Human intention prediction

Pedestrians have subjective intentions to guide themselves to reach their expected goals. Recently, some researchers have begun to research the endpoint prediction of pedestrians. Mangalam et al. (2020) used the CVAE module to predict the endpoint information and then predicted the complete trajectory. Different from the previous model, Lerner et al. (2007) used the bidirectional trajectory fitting method to predict the complete trajectory in the stage of generating the complete trajectory. Zhao et al. (2021) propose to set up multiple candidate endpoints in the region where pedestrians are likely to reach and score different candidate endpoints based on pedestrian characteristics. Gu et al. (2021) improved TNT (Zhao et al., 2021) and proposed a trajectory prediction method without pre-defining candidate targets. It dramatically improves the performance of target estimation without relying on heuristic predefined target quality. Unlike previous work that only modeled a single long-term objective, Robicquet et al. (2016) proposed a step-wise objective-driven network for trajectory prediction that evaluates and uses the goal at multiple time scales.

## 3 Method

**Figure 2:**
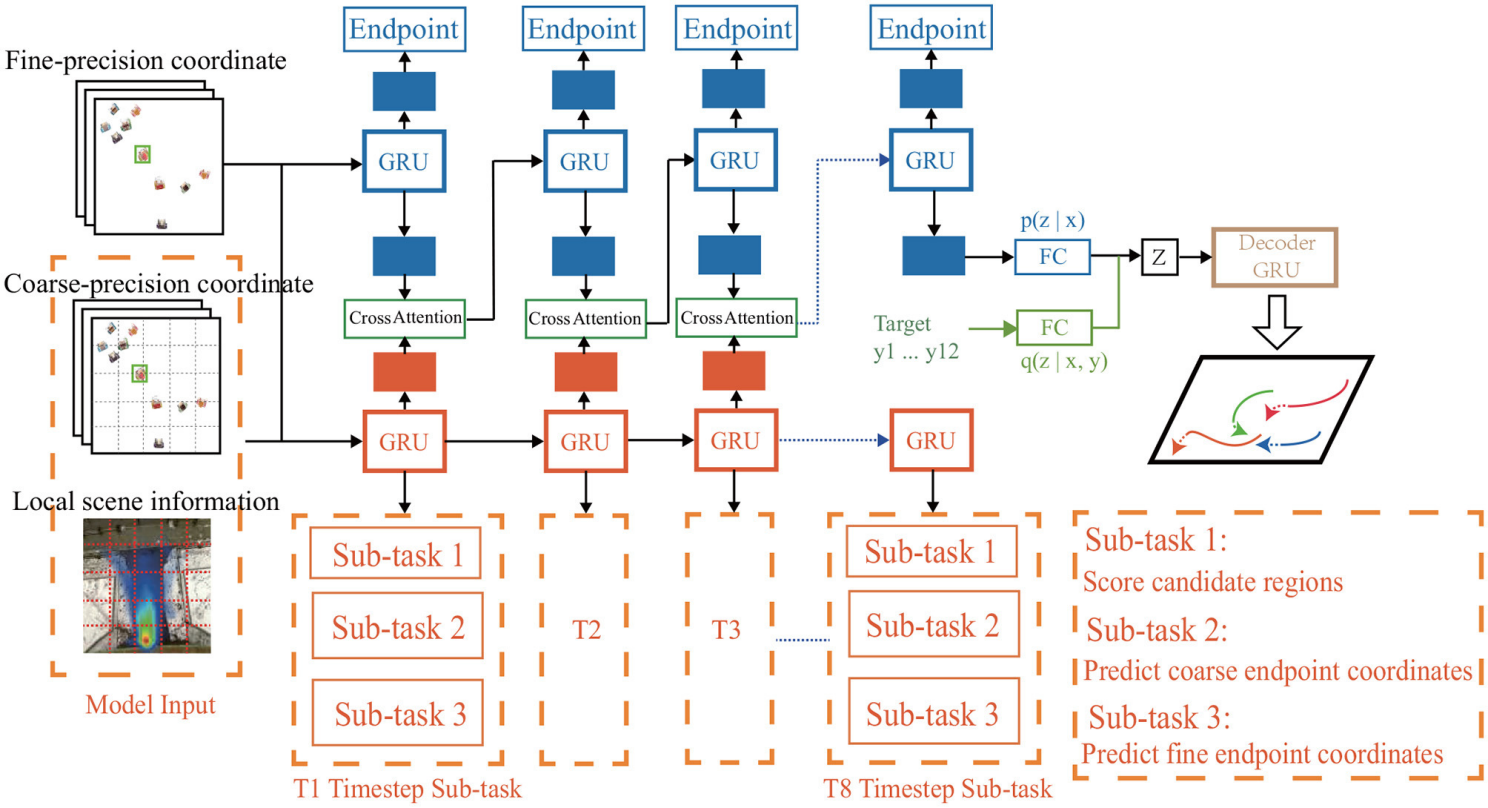
Overview of our proposed DTDNet. Take fine-precision, coarse-percision pedestrian coordinate and local scene information as input, the DTDNet is consisted of three parts: a motion pattern encoding module based on pedestrian historical trajectories (blue), a dynamic multi-task intent analysis module based on multi-precision feature input (orange), a multi-modal trajectory decoding module based on the CVAE freamwork (green and brown). Green part in the CVAE module is used only in the training stage.

In this section, we introduce structure of our DTDNet model, as shown in Figure 2. At first, we present the construction of multi-precision data. Then we discuss the three sub-networks of DTDNet: the motion pattern encoding module, multi-precision pedestrian intention analysis module and trajectory decoding module.

### 3.1 Formulations

We assume that there are *N* pedestrians in the scene *I*, the position coordinates of pedestrian *i* at time step *t* is denoted as $P_{i}^{t}=\left(x_{i}^{t},y_{i}^{t}\right)$. Our model uses historical trajectories $P_{i\_h}=\{P_{i}^{t},t\in [1,T_{obs}]\}$ to predict the future locations ${\hat{P}}_{i\text{\_}f}=\left\{\hat{P_{i}^{t}},t\in \left[T_{obs+1},T_{pre}\right]\right\}$ and minimize the distance between prediction and future trajectory $\overset{T_{pre}}{\underset{t=T_{obs+1}}{\sum }}\overset{N}{\underset{\text{i}=1}{\sum }}∥{\hat{P}}_{i}^{t}-P_{i}^{t}{∥}_{2}$.

### 3.2 Multi-precision data construction

We get three kinds of data for the model to perform the multi-precision modeling, namely fine-precision coordinates, coarse-precision coordinates, and dynamic local scene information.

#### 3.2.1 Coarse precision coordinate generation

A schematic diagram of coarse-precision coordinates is shown on the left in Figure 2, the model divides the global scene into multiple sub-regions. The region coordinates are the input coarse-precision coordinates, which retain the physical information of the scene location and are easy to combine with the scene information.

First, we collect coordinate ranges (*x*_*min*_, *x*_*max*_, *y*_*min*_, *y*_*max*_) of different scenes based on the training data. Following the principle of equal spacing, we get the segmentation space of each region according to the set division resolution *R* = *m*×*n*. Furthermore, we could use the pedestrian's current position *P*_*i*_, the coordinate range of the scene (*x*_*min*_, *x*_*max*_, *y*_*min*_, *y*_*max*_), and the length of the region to calculate the coarse precision coordinates. By using Algorithm 1, we could get the pedestrians' coarse precision coordinates *PR* as shown in Algorithm 1.

**Algorithm 1:**
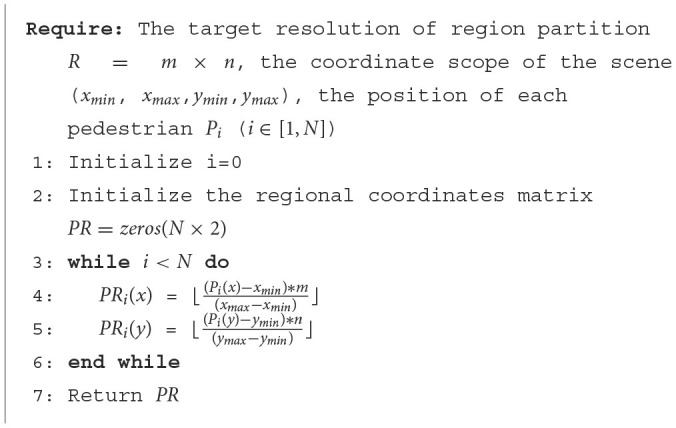
Strategy of coarse-precision coordinate generation.

#### 3.2.2 Fine precision coordinate generation

After obtaining the coarse-precision coordinates of pedestrians, we perform data pre-processing on both fine-precision coordinates and coarse-precision coordinates. To increase the generation capability of the model, we set the position (*x*_*T*_*obs*__, *y*_*T*_*obs*__) of the target pedestrian at the last observation time step as the origin, and convert the absolute position into relative position according to the position of origin.

We adopt the same data pre-processing method as Trajectron++ (Salzmann et al., 2020). In addition to the position coordinates, the input data also uses the first-order derivation and second-order derivation of position to calculate the speed information and acceleration information in both *x* and *y* direction. And we augment the training dataset by rotating all trajectories every 15 degrees around the origin point.

#### 3.2.3 Dynamic scene information

Most existing methods use semantic segmentation of the scene image to model scene information. Although semantic segmentation information has proved useful in 3D stereo reconstruction and other fields, this information is ambiguous and lacks the interaction semantics between scenes and pedestrians. For example, the lawn beside the road is defined the same as the lawn in the park. However, the lawn in the park is allowed for pedestrians to walk, and the roadside lawn is generally prohibited for pedestrians. The two have the same semantic information, but different social rules.

To solve the ambiguity of pedestrian interaction with semantic segmentation and make the scene information guide pedestrian future movement more accurately, DTDNet uses the method of STHGLU (Wang et al., 2022) to get the probability heatmap of each scene generated from historical trajectory collections. This method could provide the distribution of pedestrian movable area and the corresponding probability information. Coarse-precision coordinates keeps the spatial location information of the scene, combined with the regional information to get the local scene information.

Assuming that the coarse precision of the scene is *R* = *m*×*n*, we divide each sub-region with the precision of 9 × 9, and obtain the global scene information with the precision of *R* = 81 × *m*×*n*. At each moment, the model dynamically models the local scene *s* based on the pedestrian coarse-precision coordinate, provides information to guide the pedestrian future movement and avoid the pedestrian moving into the unreasonable area.

### 3.3 Motion pattern encoding sub-network

As shown in the upper blue part of Figure 2, the backbone of motion pattern encoding module is GRU, which inputs the fine-precision coordinates of pedestrians to model the motion pattern feature of pedestrians.

In Equation 1, we encode three input trajectory data including the position *x*^*t*^, *y*^*t*^, velocity Δ*x*^*t*^, Δ*y*^*t*^ and acceleration *ax*^*t*^, *ay*^*t*^ to the pedestrian motion hidden representation *e*^*t*^. In addition to the pedestrian motion state *e*^*t*^, as shown in Equation 2, the model includes the pedestrian target intent vector *g*^*t*^. At each moment, the endpoint decoding module uses the MLP as *f*_*goal*_ to map the output of GRU to the endpoint coordinates ${\hat{p}}_{g}^{t}$ of pedestrian, as shown in Equation 3. The goal prediction is trained with Lossdes, as shown in Equation 4. The goal prediction is trained with *Loss*_*des*_, which is the distance between the real and the predict goal. Generation of the target intention vector *g*^*t*^ from *h*^*t*^ will be introduced in detail in Section 3.4.2.


(1) $$\begin{array}{l} e^{t}=f_{e}\left(x^{t},y^{t},{\Delta x}^{t},{\Delta y}^{t},{ax}^{t},{ay}^{t};W_{e}\right) \end{array}$$

(2) $$\begin{array}{l} h^{t}=GRU\left(h^{t-1},e_{i}^{t},g^{t};W_{GRU}\right) \end{array}$$

(3) $$\begin{array}{l} {\hat{p}}_{g}^{t}=f_{goal}\left(h^{t};W_{goal}\right) \end{array}$$

(4) $$\begin{array}{l} Loss_{des}=MSE\left({\hat{p}}_{g}^{t},p_{g}\right) \end{array}$$

### 3.4 Dynamic pedestrian target prediction

#### 3.4.1 Multi-precision pedestrian intention analysis sub-network

In the model, the output *h*^*t*^ is used to predict the pedestrian target coordinates at each time step, using the mean square error as loss can not guarantee complete converge at. In order to model the pedestrian's target intention and achieve a better convergence effect, we design a pedestrian dynamic intent prediction sub-network to update the pedestrian's intent dynamically.

The model input of the sub-network consists of three parts: the fine-precision coordinate *p*_*f*_, the coarse-precision coordinate *p*_*c*_, the scene information *s*. It is the same as Equation 1, the multi-layer perceptron encodes the fine-precision *p*_*f*_ and coarse-precision *p*_*c*_ coordinate and obtains embeddings *e*_*f*_ and *e*_*c*_, respectively. As shown in Equation 5, the model uses the convolutional neural network (CNN) to encode the local scene information *s*^*t*^ to obtain $h_{s}^{t}$.


(5) $$\begin{array}{l} h_{s}^{t}=CNN\left(s^{t};W_{cnn}\right) \end{array}$$

In order to model the time series features and fuse them with the modeling information of the main network, we also use GRU to model the sequence of three kinds of information input by the sub-network. As shown in Equation 6, the input of the GRU model of the sub-network contains $e_{f}^{t}$, $e_{c}^{t}$, $h_{s}^{t}$ three dimensions of information, the output $h_{sub}^{t}$ is the intent embedding predicted by the sub-network at time *t*, and *W*_*GRUsub*_ is the training parameters.


(6) $$\begin{array}{l} h_{sub}^{t}=\underset{sub}{GRU}\left(h_{sub}^{t-1},e_{f}^{t},e_{c}^{t},h_{s}^{t};W_{\underset{sub}{GRU}}\right) \end{array}$$

#### 3.4.2 Multi-precision pedestrian intention analysis sub-tasks

To extract the pedestrian intention feature, in addition to predicting the fine-precision coordinates of the target coordinate, DTDNet proposes two additional sub-tasks to model the pedestrian intent information, namely predicting the coarse-precision endpoint region and score the pedestrian intent destination region.

The first sub-tasks is shown in Equation 7. The model uses the MLP *f*_*f*_ to map the pedestrian motion intention embedding $h_{sub}^{t}$ to predict the fine-precision coordinates of the pedestrian intention, where *W*_*f*_ are trainable parameters.


(7) $$\begin{array}{l} {\hat{p}}_{f}=f_{f}\left(h_{sub}^{t};W_{f}\right) \end{array}$$

The second sub-tasks is shown in Equation 8. The model uses the MLP *f*_*c*_ to map the pedestrian motion intention vector $h_{sub}^{t}$ to predict the coarse-precision coordinates of the pedestrian's endpoint, where *W*_*c*_ are the model update parameters.


(8) $$\begin{array}{l} {\hat{p}}_{c}=f_{c}\left(h_{sub}^{t};W_{c}\right) \end{array}$$

The third sub-task is to estimate the likelihood of all sub-regions. First, the model uses the MLP *f*_*score*_ to map $h_{sub}^{t}$, where *W*_*score*_ are the model update parameters. Then uses the Softmax function to score *R* = *m*×*n* sub-regions in the scene, as shown in Equation 9. Because there is only one ground truth region, we set the score of the true region to 1 and the scores of other regions to 0.


(9) $$\begin{array}{l} score=Softmax\left(f_{score}\left(h_{sub}^{t};W_{score}\right)\right) \end{array}$$

Through the above introduction, the loss function of the sub-network consists of three parts as shown in Equation 10. Where $\hat{p}$ is the endpoint coordinate predicted by the model, *p* is the actual endpoint coordinate, *score* is the region scoring result, the *label* is the actual region scoring label, and *L*_*CE*_ is the cross-entropy function.


(10) $$\begin{array}{r} Loss_{sub}=RMSE\left({\hat{p}}_{f},p_{f}\right)+RMSE\left({\hat{p}}_{c},p_{c}\right)\\ +L_{CE}(score,label) \end{array}$$

However, since the current sub-network and the main network are decoupled, the main network cannot use the sub-networks loss function to assist in the model update. In order to use the back-propagation of the model to update the two networks synchronously, we design two network fusion schemes to couple the two parts of the network.

The first method is to fuse the motion state of the main network with the important scene information selected by the sub-network. The sub-network of the model scores the importance of *m*×*n* sub-regions at each moment and selects the Top K with the highest scores. The target sub-region is used as the key region, and the CNN shown in Equation 5 encodes the selected K regions, respectively.


(11) $$\begin{array}{l} h_{s}^{t}=\overset{K}{\underset{j=1}{\sum }}score_{j}\times h_{s}^{j} \end{array}$$

After encoding K regions, the model uses Equation 11 to fuse K scene information to obtain the crucial regional information that pedestrians need to consider. Finally, the multi-attention mechanism and residual connection are used to combine the two networks to get the target intention vector *g*^*t*^.


(12) $$\begin{array}{l} s_{r}=Softmax\left(\frac{<W_{Q}h_{r}^{t},W_{K}h_{s,r}^{t}>}{\sqrt{D}}\right) \end{array}$$

(13) $$\begin{array}{l} g^{t}=\sum_{r\in \left\{1,...,p\right\}}s_{r}\cdot \left(W_{V}h_{s,r}^{t}\right)+h^{t} \end{array}$$

Where < ·, ·> is the inner product operator, and *r*∈{1, …, *p*}, *W*_*Q*_, *W*_*K*_ and *W*_*V*_ are trainable parameters, *h*^*t*^ is the output of the motion encoding network GRU of time step t, *D* is the embedding dimension of *h*^*t*^, *p* is the number of heads in the multi-head attention mechanism, *s*_*r*_ is the attention score, and *g*^*t*^ is the target intent embedding.

The fusion method introduced in Algorithm 1 directly combines K important scene information, which may introduce excessively artificially set rule information. It is difficult to determine the optimal value of parameter K. Therefore, we attempt to directly fuse the output $h_{sub}^{t}$ of the sub-network with the GRU output *h*^*t*^ of the main network using the attention mechanism introduced in Equations 12, 13.

### 3.5 Trajectory decoding sub-network

This sub-network utilizes CVAE based framework to generate multi-modal trajectories. CVAE framework is composed by an encoding module and a decoding module. The encoding network is further divided into a recognition distribution network *q*_ψ_(*z*|**P_h_**, **P_f_**) and a prior distribution network *p*_θ_(*z*|**P_h_**) given future ground truth trajectory as $P_{f}=\{P^{t},t\in [T_{obs+1},T_{pre}]\}$.

As shown in Equation 14, the model encodes the pedestrian historical and future motion feature, and generates the mean μ and variance σ corresponding to a Gaussian distribution, and samples high-dimensional latent variable *z* from Gaussian distribution *N*(μ, σ). Then combines the sampled high-dimensional latent variable *z* with the GRU output *h*^*t*^ to obtain the hidden state $h_{dec}^{t}$, and iterate the hidden state at each time step, as shown in Equations 15, 16. Finally use the decoding module Equation 17 to predict the complete future trajectory.


(14) $$\mu ,\sigma =q_{\psi }(z|P_{h},P_{f}),z\sim N(\mu ,\sigma )$$

(15) $$\begin{array}{l} h_{dec}^{T_{obs}}=f_{mlp}\left(h^{T_{obs}}⊕z;W_{mlp}\right) \end{array}$$

(16) $$\begin{array}{l} h_{dec}^{t+1}=D-GRU\left(h_{dec}^{t},f_{pred}\left({\hat{x}}^{t},{ŷ}^{t};W_{pred}\right)\right) \end{array}$$

(17) $$\begin{array}{l} {\hat{x}}^{t+1},{ŷ}^{t+1}=f_{decoder}\left(h_{dec}^{t+1};W_{decoder}\right) \end{array}$$

Where *q*_ψ_, *f*_*mlp*_,*f*_*pred*_,*f*_*decoder*_ are implemented as MLPs, and ⊕ represents the concatenate operation. $h_{dec}^{T_{obs}}$ represents the initial embedding of decoder GRU(D-GRU), *h*^*obs*^ is the motion information of the pedestrian at time *T*_*obs*_, *z* represents the latent variable generated by the CVAE framework; ${\hat{x}}^{t}$,ŷ^*t*^ represents the pedestrian position predicted by the model at time step *t*.

In the testing phase, the latent variable *z* is directly sampled from *p*_θ_(*z*|**P_h_**), and the recognition distribution is not calculated. We use *KL* divergence to make sure that prior distribution is same with the recognition distribution in the training stage, as shown in Equation 18. Finally, the model is trained end-to-end from loss *Loss*_*variety*_, which is composed by the KL-divergence, sub-tasks loss, goal prediction loss, and the distance between the best prediction and the future trajectory, as shown in Equation 19.


(18) $$Loss_{KLD}=KLD(q_{\psi }(z|P_{h},P_{f}),p_{\theta }(z|P_{h}))$$

(19) $$\begin{array}{r} Loss_{variety}=\underset{k}{min}\sum_{t=T_{obs+1}}^{T_{pre}}{\left\|{\hat{p}}_{k}^{t}-p^{t}\right\|}_{1}+Loss_{des}\\ +Loss_{KLD}+Loss_{sub} \end{array}$$

## 4 Experiments and results

Datasets: We evaluate the performance of our model and report results on two real-world public datasets: ETH-UCY Dataset (Pellegrini et al., 2009; Dendorfer et al., 2021) and Stanford Drone Dataset (Shi et al., 2021). **ETH-UCY** contains five subsets: ETH, HOTEL, UNIV, ZARA1, ZARA2. It contains 1,536 pedestrians and introduces interactions like group interactions, collision avoidance. We follow the experimental settings in Trajectron++ (Yu et al., 2020), which convert the data to the world coordinate system and split them into 8 s segments (20 time steps). We use historical 3.2 s (eight time steps) to predict the future 4.8 s (12 time steps). **Stanford Drone Dataset** contains 20 scenes. We use the data released by NMMP (Tao et al., 2020), whose coordinates of trajectories are provided in pixels, and the experimental settings are the same as ETH-UCY. For the ETH-UCY and Stanford Drone Dataset, we use the leave-one-out evaluation strategy to test different models.

Implementation details: We train our models with Adam optimizer, batch size 64, learning rate 0.0001 on a single NVIDIA Tesla T4 GPU. In coarse-precision modeling, we adopt different partitioning strategies. We divide ETH-UCY into 5 × 5 regions, and Stanford Drone Dataset into 9 × 9 regions. The resolution of scene information for each sub-region is 9 × 9. MLP and GRU hidden layer dimension are set to 256. The dimension of latent variable *z* is 64, which is sampled from a CVAE framework generated distribution. The hyper-parameter of variety loss weight is set to 20.

### 4.1 Quantitative evaluation

We compare our method with seven state-of-the-art methods, including PMP-NMMP, Social-STGCNN, STAR, PECNET, Trajectron++. The results are shown in Table 1, which are evaluated with the ADE and FDE metrics. The results indicate that our method significantly outperforms all the competing methods on the ETH and UCY datasets. Our method outperforms Agentformer (Yuan et al., 2021) by 17.4% on the ADE metric, and on the FDE metric, our method outperforms Agentformer by 7.7%.

**Table 1:** Quantitative results of all the previous state-of-the-art methods and our model on ETH-UCY.

**Method**	**ETH**	**HOTEL**	**UNIV**	**ZARA1**	**ZARA2**	**AVG**
PMP-NMMP (Tao et al., 2020)	0.61/1.08	0.33/0.63	0.52/1.11	0.32/0.66	0.29/0.61	0.41/0.82
Social-STGCNN (Hu et al., 2020)	0.64/1.11	0.49/0.85	0.44/0.79	0.34/0.48	0.30/0.48	0.44/0.75
*STAR* (Yuan et al., 2021)	**0.36/0.65**	0.17/0.36	0.31/0.62	0.26/0.55	0.22/0.46	0.26/0.53
*PECNet* (Mangalam et al., 2020)	0.54/0.87	0.18/0.24	0.35/0.60	0.22/0.39	0.17/0.30	0.29/0.48
Trajectron++ (Yu et al., 2020)	0.43/0.86	**0.12/0.19**	0.22/**0.43**	0.17/0.32	0.12/0.25	0.21/0.41
MG-GAN (Dendorfer et al., 2021)	0.47/0.91	0.14/0.24	0.54/1.07	0.36/0.73	0.29/0.60	0.36/0.71
*SGCN* (Shi et al., 2021)	0.63/1.03	0.32/0.55	0.37/0.70	0.29/0.53	0.25/0.45	0.37/0.65
*Agentformer* (Yuan et al., 2021)	0.45/0.75	0.14/0.22	0.25/0.45	0.18/0.30	0.14/0.24	0.23/0.39
DTDNet (No sub-tasks)	0.38/0.69	0.13/0.24	0.23/0.47	0.13/0.27	0.12/0.24	0.20/0.38
DTDNet (Ours)	0.37/0.67	0.13/0.23	**0.21**/0.44	**0.13/0.26**	**0.12/0.23**	**0.19/0.36**

We calculate the metrics for *T*_*obs*_ = 8 (3.2s) and *T*_*pre*_ = 12 (4.8 s) (best of 20 samples). The bold value indicates the best result.

To compare the results of deterministic sampling, we compared the past three models, namely STGAT, STAR, and Trajectron++. The experimental results are shown in Table 2. Although our method is consistent with Trajectron++ in ADE metrics, our method is superior to Trajectorn++ by 12.6% in FDE, which shows that the intent prediction module has played a role, and pedestrians' intent coordinates could be predicted more accurately.

**Table 2:** Quantitative results of all the previous state-of-the-art methods and our model on ETH-UCY.

**Method**	**ETH**	**HOTEL**	**UNIV**	**ZARA1**	**ZARA2**	**AVG**
*STGAT* (Mohamed et al., 2020)	0.88/1.66	0.56/1.15	0.51/1.13	0.41/0.91	0.31/0.68	0.51/1.11
*STAR* (Yuan et al., 2021)	**0.56/1.11**	0.26/0.50	0.52/1.15	0.41/0.90	0.31/0.71	0.41/0.87
Trajectron++ (Yu et al., 2020)	0.71/1.68	**0.22/0.46**	**0.41**/1.07	0.30/0.77	0.23/0.59	0.37/0.95
DTDNet (Ours)	0.63/1.42	0.25/0.51	0.43/**1.01**	**0.26/0.63**	**0.24/0.57**	**0.36/0.83**

We calculate the metrics for *T*_*obs*_ = 8 (3.2 s) and *T*_*pre*_ = 12 (4.8 s) (one sample). The bold value indicates the best result.

Table 3 shows the experimental results of Stanford Drone Dataset. The scenes of Stanford Drone Dataset are rich and various, and our model performs better than all previous works on this dataset. We outperform the best Trajectron++ model on the ADE metrics by 7.1%, and in the FDE metrics, our method outperforms the PECNet model by 3.1%. It means that our model has a better ability in the migration of different scenes.

**Table 3:** Quantitative comparison on Stanford Drone Dataset.

**Method**	**ADE**	**FDE**
Sophie (Vemula et al., 2018)	16.3	29.4
PMP-NMMPN (Tao et al., 2020)	14.7	26.7
STGAT (Mohamed et al., 2020)	14.2	26.7
MG-GAN (Dendorfer et al., 2021)	13.6	25.8
Trajectron++ (Yu et al., 2020)	9.9	16.8
*PECNet* (Mangalam et al., 2020)	10.0	15.9
DTDNet (Ours)	**9.2**	**15.4**

Given previous 3.2 s, predicting future 4.8 s. ADE/FDE is reported in pixels (20 samples). The bold value indicates the best result.

### 4.2 Ablation study

To verify the role of the auxiliary loss function in the sub-tasks, we designed an ablation experiment on ETH-UCY dataset for comparison in the last two lines of Table 1. The ablation model still retains local scene information and coarse-precision coordinates but does not add the loss function for auxiliary sub-tasks updates. Compared with the ablation model, the whole model can improve the ADE and FDE metrics by 5.0 and 5.6%, respectively.

To evaluate the promotion effect of the three sub-tasks on pedestrian intent prediction, as shown in Table 4, we designed four ablation models on SDD dataset for comparative experiments: (1) Replace the CVAE module with Gaussian noise sampling, (2) without the sub-task of scene scoring, (3) without the coarse-precision prediction sub-task, (4) without the fine-precision prediction sub-task. It shows that the fine-precision prediction task is still the most effective task that affects the trajectory prediction results most significantly. The coarse-precision prediction and scene scoring tasks also could improve the trajectory prediction effect. Our model does not take any pedestrian interaction information into consideration, which shows that only using pedestrian motion features and scene information could achieve sota results.

**Table 4:** Ablation study of DTDNet structure on Stanford Drone Dataset.

**Method**	**ADE**	**FDE**
*No CVAE module*	9.7	16.4
*No scene scoringmodule*	9.4	15.8
*No coarse precision loss function*	9.5	15.9
*No fine precision loss function*	9.6	16.1
DTDNet (Ours)	**9.2**	**15.4**

Given previous 3.2 s, predicting future 4.8 s. ADE/FDE is reported in pixels (choose the best from 20 samples). The bold value indicates the best result.

To evaluate the effectiveness of the sub-tasks and choose an appropriate region division accuracy, we conduct experiments in Tables 5, 6. In Table 5, we conduct experiments with different coarse precision settings on the SDD dataset, and the ADE/FDE results show that the 9 × 9 precision division results are better than other precision settings. In Table 6, we evaluated the recall for the important region scoring sub-tasks at time step *T*_8_ and compared the effects of different region division accuracy and different recall numbers. *TP* is the number of target regions recalled by the model, *P* is the number of samples in the test experiment, each sample has only one target region, and *P*_*recall*_ is the recall rate, as shown in Equation 20.


(20) $$\begin{array}{l} P_{\text{recall}}=\frac{TP}{P}\times 100\% \end{array}$$

**Table 5:** Ablation study of different coarse precisions on Stanford Drone Dataset (ADE/FDE is reported).

**Precision**	**ADE**	**FDE**
5 × 5	9.4	15.8
9 × 9	**9.2**	**15.4**
15 × 15	9.3	15.5

The bold value indicates the best result.

**Table 6:** Relationship between recall rate *P* and recall number *k* under different precisions on Stanford Drone Dataset.

***Precision***	**1**	**2**	**3**	**4**	**5**	**6**
5 × 5	61.8%	84.6%	91.4%	96.9%	98.3%	99.1%
9 × 9	**68.6%**	**89.3%**	**95.1%**	**98.3%**	**99.1%**	**99.6%**
15 × 15	67.2%	88.1%	94.2%	98.2%	99.0%	99.4%

The bold value indicates the best result.

Table 6 shows that the model recalls the Top 1 scored region, and the recall rate of the target area is more than 60%. When the recall number is 6, the recall rate of the target region is close to 100%. The regional scoring task can identify important areas and predict the target region of pedestrians with better accuracy. Table 6 shows that the recall rate of the model in the 9 × 9 precision are better than the 5 × 5 or 15 × 15 precision. This result is consistent with the results in Table 5, so we set the coarse precision size to 9 × 9 on dataset with a larger scene.

### 4.3 Qualitative evaluation

#### 4.3.1 Visualization of the DTDNet and ground truth

**Figure 3:**
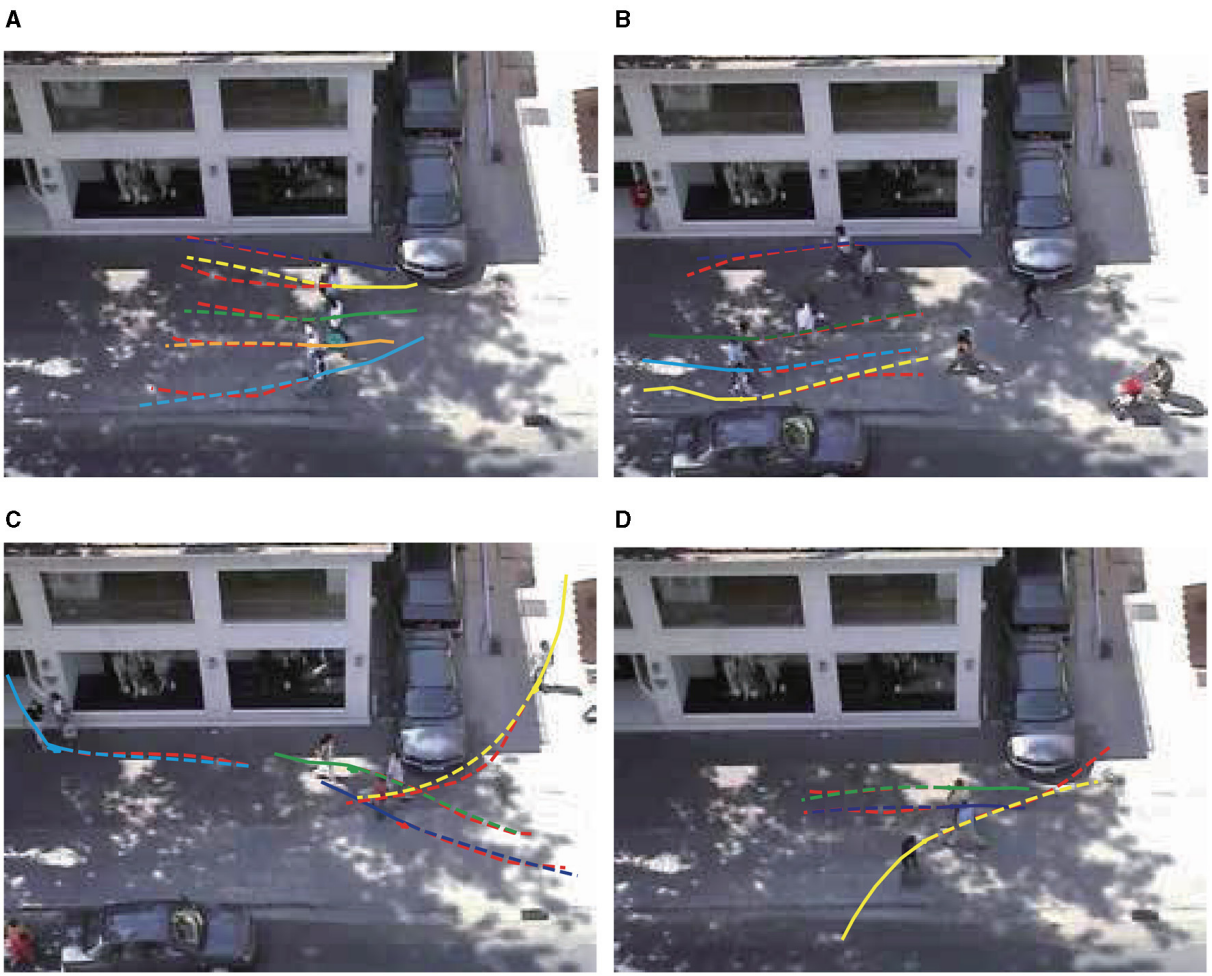
**(A–D)** Qualitative analysis of DTDNet. For a better view, only part of the pedestrians in the scene is presented. The illustration scenes are selected from ZARA1. Observed trajectories are shown as solid lines, and the predicted trajectories are shown as dashed lines. The red line represents the true trajectory.

We select two motion modes for display: group motion and pedestrian motion to avoid collision. In Figures 3A, B, multiple groups of pedestrians are moving in the same direction, and the results predicted by our model almost completely fit the actual red trajectories. In Figures 3C, D, the pedestrian motion trajectory avoids collision with surrounding pedestrians and obstacles. Our model predicts the pedestrian's turning motion intention and effectively predicts the pedestrian's offset angle, avoids collision with vehicles and passing pedestrians.

#### 4.3.2 Visualization of the trajectory distribution

**Figure 4:**
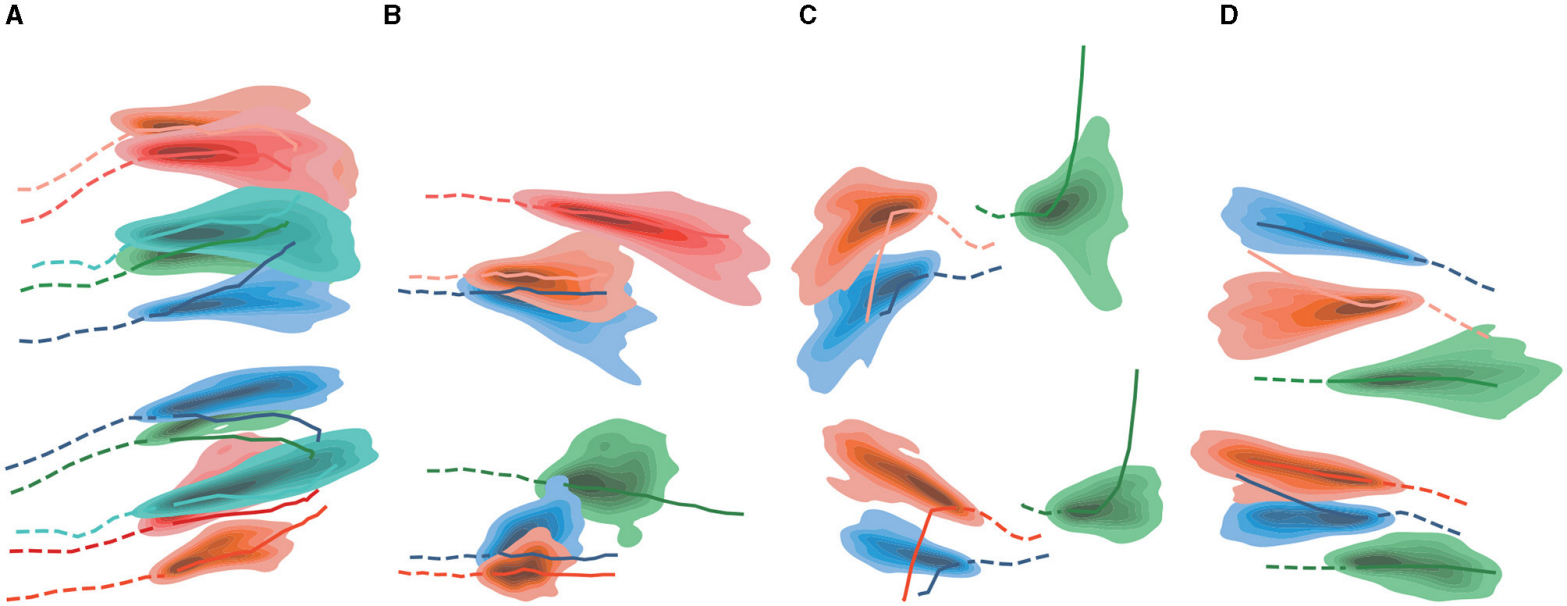
**(A–D)** Qualitative analysis of DTDNet and Social-STGCNN. Upper ones are from DTDNet, lower ones are from Social-STGCNN.

As shown in the Figure 4, we compare our model (DTDNet) with Social-STGCNN in four different scenarios selected from ETH, HOTEL, ZARA1 and ZARA2 dataset. The dashed line represents the observed trajectory, and the solid line represents ground truth of the prediction and the color density is the predicted trajectory distribution. Figure 4A shows that the future trajectories of the two pedestrians above are slightly shifted downward, DTDNet model predicts the same trajectory distribution, but Social-STGCNN predicts that the pedestrians are still going straight. As shown in Figure 4B, compared with Social-STGCNN, DTDNet can predict the pedestrian's speed and the pedestrian's endpoint more accurately, so it can cover the true trajectory of the pedestrian. We could even predict multiple distribution trends in cases where there may be many likely future trajectories, and our generation framework does not have a mode collapse problem like other methods. As shown in Figure 4C, taking the green trajectory in the figure as example, DTDNet not only predicts the movement of turning upward, but also predicts the trend of downward turning. However, the prediction effect of the model also has certain shortcomings. As shown in Figure 4D, when pedestrians perform a sudden turning in the prediction time region, existing methods cannot predict the turning trend successfully. In future, we will try to introduce interactive information between dynamic obstacles in the predicting period to explore this problem.

#### 4.3.3 Visualization of intention prediction

**Figure 5:**
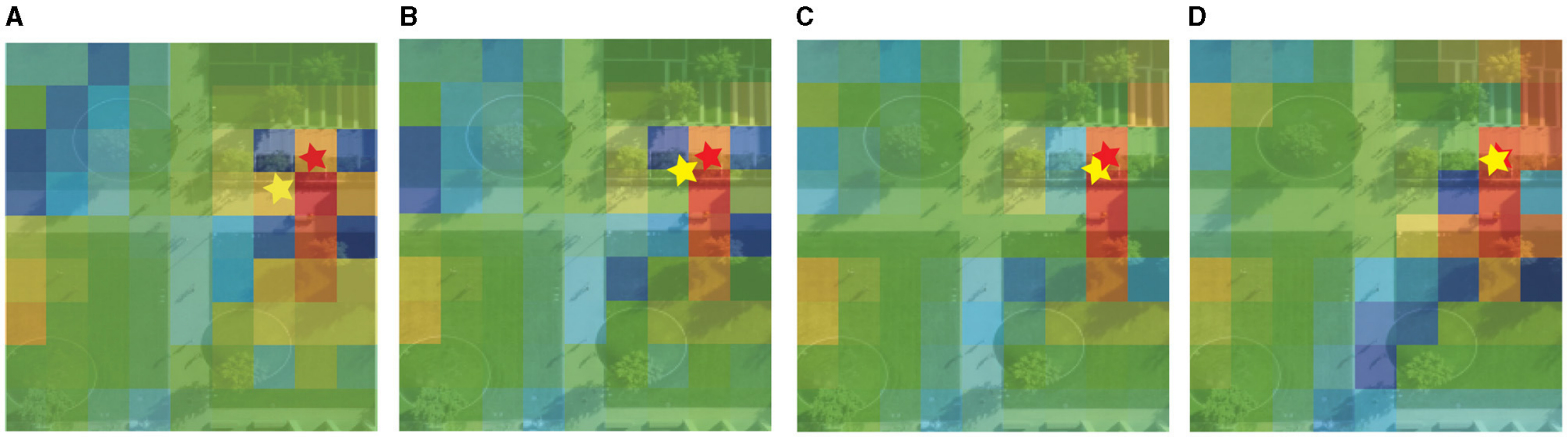
Visualization of dynamic intent prediction by DTDNet. Red star is the future endpoint, and yellow star represents the predicted target. Color of each sub-region represents the scene importance score. Red is the highest, green is the middle and blue is the lowest score. **(A)** T1. **(B)** T3. **(C)** T5. **(D)** T8.

To exhibit the dynamic prediction of pedestrian intent coordinate, we select a scene from the Stanford Drone Dataset and visualize the dynamic pedestrian intent and regional score predicted by the model in Figure 5. The red star represents the future target endpoint, and the yellow star represents the predicted target coordinate at different time steps, the color of each sub-region represents the magnitude of the scene importance score, and the red region represents the high score. In Figure 5, four time step results of pedestrian movement and divide the scene into 81 sub-regions according to the precision of 9 × 9. The model dynamically predicts pedestrian intent coordinates and the importance score of the scene. As the pedestrian moves, the target coordinate of the yellow star predicted by the model gradually approaches the real target. The importance score of the region near the actual location gradually increases. The color of the visualization gradually turns red, such as the region where the red star is located by the yellow at time *T*_1_ in Figure 5A becomes red at time *T*_8_ in Figure 5D. The number of the red regions near the finish area also increases significantly.

## 5 Conclusion

In this work, we propose DTDNet, a Dynamic Target Driven Network for pedestrian trajectory prediction. Different from previous models that predict a fixed endpoint, DTDNet is designed to model the intention of a pedestrian dynamically with a hidden representation. This hidden representation could jointly represents mixture information of intention. We also introduce a multi-precision data representation method and three sub-tasks to analyze pedestrians motion intentions from different precision feature. The three sub-tasks are proved helpful to make sure the hidden representation could converge and be useful to the intention representation at each time step. Our proposed model is a superior to the baseline models in quantitative metrics on two publicly available datasets. Qualitative experiments show that our model could predict pedestrian intention accurately and dynamically. In the future, research should consider the potential effects of bringing related subtasks to help the network hidden representation of pedestrian converge better and add more supervision to the feature. Furthermore, the dynamic modeling of intentions at each timestep, along with predictions, could benefit from a more complicate network architecture that incorporates the modeling of complex interactions among moving objects within the scene to distill involved information.

## Data availability statement

Publicly available datasets were analyzed in this study. This data can be found at: https://github.com/StanfordASL/Trajectron-plus-plus/tree/master/experiments/pedestrians/raw.

## Author contributions

SL: Conceptualization, Methodology, Supervision, Writing – review & editing. JS: Conceptualization, Methodology, Software, Writing – original draft. PY: Methodology, Validation, Visualization, Writing – review & editing. YZ: Methodology, Validation, Visualization, Writing – review & editing, Software. TM: Conceptualization, Methodology, Project administration, Supervision, Writing – review & editing. ZW: Project administration, Supervision, Writing – review & editing.
